# Decidual/placental and first trimester plasma levels of hsa-miR-199a-3p|hsa-miR-199b-3p and hsa-miR-3150b-3p are associated with insulin secretion in pregnancy

**DOI:** 10.3389/fendo.2025.1622500

**Published:** 2025-08-19

**Authors:** Amélie Taschereau, Frédérique White, Catherine Allard, Andrea G. Edlow, François Aguet, Kristin G. Ardlie, Jose C. Florez, Pierre-Étienne Jacques, S. Ananth Karumanchi, Camille E. Powe, Luigi Bouchard, Marie-France Hivert

**Affiliations:** ^1^ Department of Biochemistry and Functional Genomics, Université de Sherbrooke, Sherbrooke, QC, Canada; ^2^ Département de Biologie, Université de Sherbrooke, Sherbrooke, QC, Canada; ^3^ Centre de Recherche du Centre Hospitalier Universitaire de Sherbrooke (CRCHUS), Sherbrooke, QC, Canada; ^4^ Department of Obstetrics and Gynecology, Massachusetts General Hospital, and Harvard Medical School, Boston, MA, United States; ^5^ Cancer Program, Broad Institute of MIT and Harvard, Cambridge, MA, United States; ^6^ GTEx Laboratory Data Analysis and Coordination Center, Broad Institute of MIT and Harvard, Cambridge, MA, United States; ^7^ Programs in Metabolism and Medical & Population Genetics, Broad Institute of MIT and Harvard, Cambridge, MA, United States; ^8^ Diabetes Unit, Endocrine Division, Department of Medicine, Massachusetts General Hospital, Boston, MA, United States; ^9^ Center for Genomic Medicine, Massachusetts General Hospital, Boston, MA, United States; ^10^ Department of Medicine, Harvard Medical School, Boston, MA, United States; ^11^ Institut de recherche sur le cancer de l’Université de Sherbrooke (IRCUS), Sherbrooke, QC, Canada; ^12^ Department of Medicine, Cedars-Sinai Medical Center, Los Angeles, CA, United States; ^13^ Department of Medical Biology, CIUSSS of Saguenay-Lac-Saint-Jean, Saguenay, QC, Canada; ^14^ Department of Population Medicine, Harvard Medical School, Harvard Pilgrim Health Care Institute, Boston, MA, United States

**Keywords:** pregnancy, placenta, insulin secretion, diabetes, microRNA

## Abstract

**Background/Aims:**

The placenta expresses and releases specific microRNAs (miRNAs) into the maternal circulation that may influence insulin secretion during pregnancy. We hypothesized that specific decidual/placental miRNAs are associated with maternal insulin secretion during pregnancy.

**Methods:**

In the Genetics of Glucose regulation in Gestation and Growth (Gen3G) prospective cohort, we estimated maternal insulin secretion using the Stumvoll first phase index derived from an oral glucose tolerance test at ~26 weeks of gestation. We quantified miRNAs by small RNA sequencing in placenta (N=435) and first trimester plasma (=422) samples. We used the Limma R package to identify miRNAs associated with the Stumvoll index (*P*<0.05). We adjusted models for Matsuda index, gravidity, maternal age, newborn sex, gestational age at sampling (first trimester plasma sampling or delivery for placenta samples), and for technical covariates (batch and run for plasma, surrogate variables for placenta).

**Results:**

Participants had a median [IQR] Stumvoll first phase index of 1112.9 [905.4 - 1284.5] in pregnancy. We identified 30 decidual/placental and 93 first trimester plasma miRNAs nominally associated with the Stumvoll first phase estimate (*P*<0.05). Lower insulin secretion was associated with lower levels of has-miR-199a-3p|has-miR-199b-3p (b=1.47 [0.10, 2.69] in placenta and b= 4.22 [0.70, 7.67] in plasma), and with higher levels of has-miR-3150b-3p (b= -6.97 [-14.39, 0.40] in placenta and b= -9.19 [-18.38, -0.60] in plasma).

**Conclusion:**

We identified hsa-miR-199a-3p|hsa-miR-199b-3p and hsa-miR-3150b-3p as differentially expressed in placenta and circulating levels associated with insulin secretion in pregnancy. Hsa-miR-199a-3p may regulate insulin secretion by modulating the expression of E-cadherin and components of the Notch signaling pathway; hsa-miR-3150b-3p may influence glucose-induced insulin secretion through interaction with phospholipase A2.

## Introduction

Gestational diabetes mellitus (GDM) affects approximately 14% of pregnancies worldwide and is associated with both short- and long-term complications for the mother and her offspring (1). Short-term complications include an increased risk of gestational hypertensive disorders, caesarean delivery, large for gestational age birth, and neonatal hypoglycemia (2, 3). In the long term, GDM is associated with an increased risk of metabolic and cardiovascular diseases in both the mother and offspring (4). GDM manifests as hyperglycemia due to excessive insulin resistance or inadequate insulin secretion or a combination of both defects. Defect in insulin secretion revealed in pregnancy affects many but not all GDM cases (5) and may be an important predictor for future maternal risk of type 2 diabetes (6).

The placenta is an important regulator of maternal metabolism during pregnancy, and its structure and function are also influenced by maternal signals during its development (7). Recent evidence suggests that the placenta may also play a role in modulating insulin secretion during pregnancy through the release of small extracellular vesicles (containing hormones and microRNAs) into the maternal circulation (8). In addition, insulin secretion increases during the first trimester, before any decline in insulin sensitivity (9). Animal studies have also shown that pancreatic beta cell proliferation during pregnancy is regulated by placental lactogen and serotonin (10, 11). However, the exact placental factors responsible for increasing insulin secretion during human pregnancy are still unknown.

MicroRNAs (miRNAs) are short non-coding, single strand RNA sequences of ~19 to 25 nucleotides, mostly implicated in protein synthesis repression (12). miRNAs are secreted into bloodstream where they are highly stable and can exert their functions in autocrine, paracrine or endocrine manners, like hormones. Placental miRNAs are mainly (not exclusively) encoded into three clusters (i.e., C19MC, C14MC, and miR-371–3 clusters), released into maternal circulation as early as the sixth week of gestation, and implicated in pregnancy processes like placentation and fetal growth (13). Circulating and placental miRNAs in pregnancy have been associated with GDM development, insulinemia and glycemia (14). These associations may be partially explained by the effects of placental miRNAs on both insulin sensitivity and secretion during pregnancy. While previous studies have reported associations between placental miRNAs and insulin sensitivity during pregnancy (15, 16), their potential role in the modulation of insulin secretion remains unclear. We therefore hypothesized that some specific decidual/placental miRNAs secreted into the maternal bloodstream contribute to modulating insulin secretion during pregnancy.

The goal of this study was to discover novel decidual/placental miRNA implicated in insulin secretion modulation during pregnancy. We investigated whether maternal insulin secretion in late second trimester was associated with decidual/placental miRNA expression levels (assessed by whole-genome miRNA sequencing), and with circulating miRNA detectable in first-trimester plasma samples, using an agnostic approach.

## Methods

### Gen3G cohort

The Genetics of Glucose regulation in Gestation and Growth (Gen3G) cohort is a prospective pregnancy and birth cohort from Sherbrooke, Canada, described previously (17). Briefly, between 2010 and 2013, we recruited 1024 women without pre-existing diabetes or overt diabetes (hemoglobin A1c ≥6.5% or glucose ≥185 mg/dL after a 50-g glucose load) at first trimester of pregnancy. Exclusion criteria were non-singleton pregnancy, alcohol use disorder, and regular use of medications affecting glucose metabolism. In addition, participants who declined biobanking of both plasma and placental samples were also excluded from the present analyses. We followed women throughout their pregnancy during which we collected data and information at first trimester (V1), in the late second trimester (V2), and at delivery. Each participant provided written informed consent according to the Declaration of Helsinki. The study protocol was approved by the ethical committees from the CHUS and the Harvard Pilgrim Health Care Institute.

### Variable collection and measurements

At V1, we collected demographic data, anthropometric measures (standardized height and weight, to calculate body mass index (BMI)), obstetric history, and blood samples. Blood samples were centrifuged, and plasma samples were aliquoted and kept at -80°C until RNA extraction.

At V2, women underwent a 75g oral glucose tolerance test (OGTT) during which we collected additional blood samples at fasting, 1h and 2h time-points to measure glucose and insulin levels. We measured glucose levels (mmol/L) immediately after blood sample collection using the hexokinase method (Roche Diagnostics; CHUS biochemistry laboratory). We aliquoted plasma samples after blood centrifugation and stored them at -80°C until insulin measurement (pg/ml), using a multiplexed particle-based flow cytometric assay (Human Milliplex MAP kits; EMD Millipore). We estimated insulin sensitivity using the Matsuda index and insulin secretion using the Stumvoll first phase index based on first phase β-cell function using 0, 60, 120 min glucose and insulin values during the 75g OGTT, without inclusion of demographic parameters into the formulas. We used the following formulas: Matsuda= 10 000/√(Gluc0 ×Ins0 ×mean glucose×mean insulin), with concentrations in glucose and insulin being expressed as mg/dL and μU/mL (18); and Stumvoll= 1194 + 4.724 Å~ Ins0 − 117.0 Å~ Gluc60 + 1.414 Å~ Ins60, with concentrations in glucose and insulin being expressed as mg/dL and pmol/L (43). We ascertained GDM using the International Association of Diabetes and Pregnancy Study Groups criteria (fasting plasma glucose ≥ 5.1mmol/L and/or 1-hour plasma glucose ≥ 10.0mmol/L and/or 2-hour plasma glucose ≥ 8.5 mmol/L) (19). Women clinically diagnosed with GDM were referred to a diabetes in pregnancy clinic managed by endocrinologists.

At delivery, we collected newborn gestational age and sex using medical records. Trained research staff collected placental tissue within 30 minutes of delivery, following a standardized protocol (17). Briefly, a 1 cm^3^ placental biopsy, including decidual tissue, was collected from the maternal facing side of the placenta within a 5 cm radius of the corresponding location of cord insertion on the other side. The biopsy sample was immediately placed in RNA-Later for at least 24 hours at 4°C, then stored at -80°C until RNA extraction.

### RNA extraction, sequencing, and quality control

Trained laboratory staff extracted total RNA from placental biopsies (15 mg) and first trimester plasma samples (500 μL) using the MirVana PARIS kit (Thermo Fisher Scientific, catalog no. AM1556) following the manufacturer’s standard procedure for tissues stored in RNAlater and liquid samples respectively. For plasma samples, total RNA was eluded in 75 μL of nuclease-free water and samples were concentrated by precipitation and resuspended in 5 μL of nuclease-free water, as previously described (20).

#### Placenta

**Figure 1:**
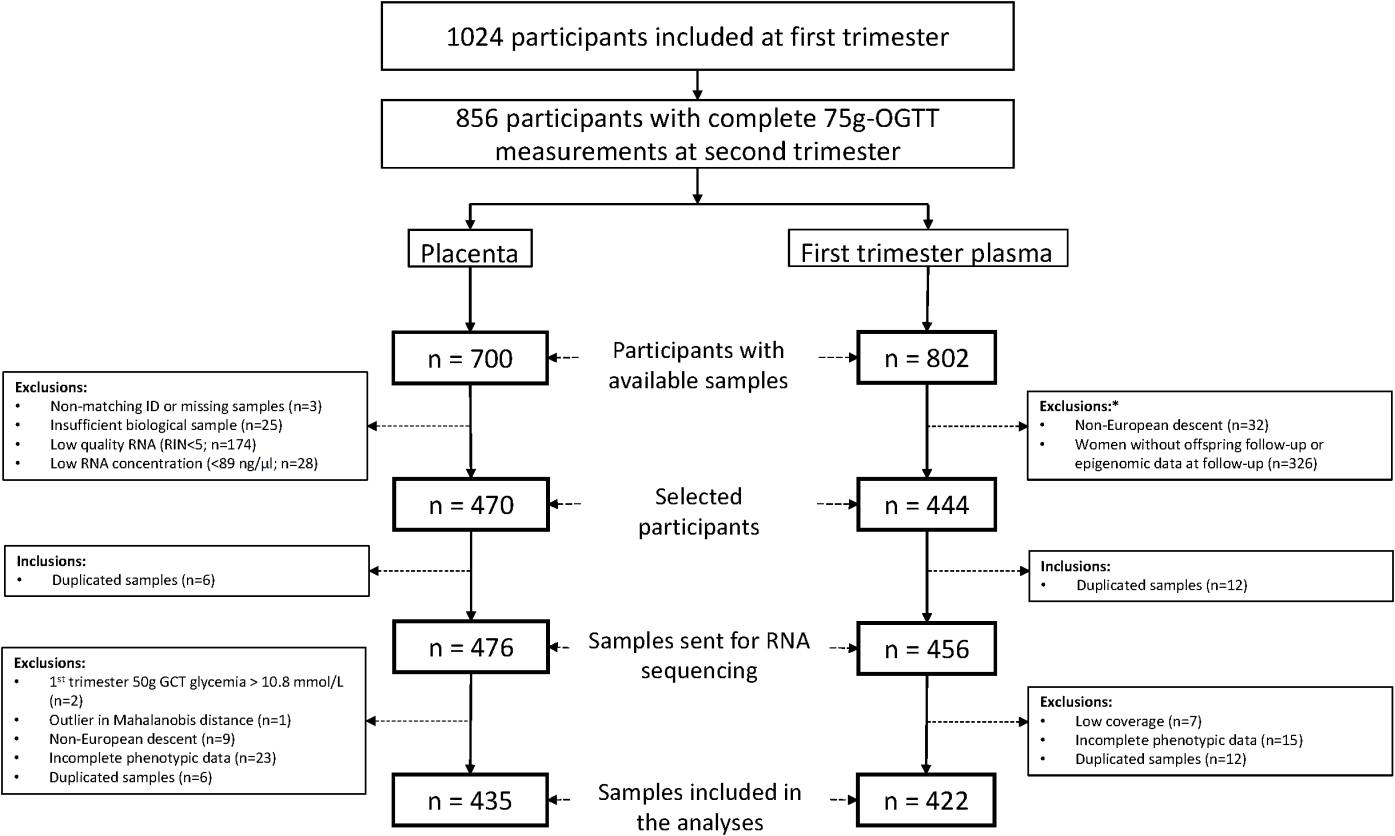
Flowchart illustrating participant selection from the Gen3G cohort for inclusion in both the placenta and first trimester plasma datasets. *Exclusion criteria for maternal plasma microRNA sequencing study. GCT, glucose challenge test; OGTT, oral glucose tolerance test; RIN, RNA integrity number.

We sent 3 μg of each sample with (RNA Integrity Number) RIN ≥ 5 and RNA concentration ≥ 89 ng/µl to Novogene for small RNA sequencing (N=476). We prepared libraries using Qiagen Small RNA Kit to generate 75-bp single end reads on NovaSeq 6000 platform. On average, we obtained 28M total reads (range 19M-60M). We excluded six duplicated samples and used Mahalanobis distance (21) on quantified miRNAs to identify and exclude one outlier sample. We additionally excluded two who had hyperglycemia detected and treated before V2, 9 non-European (outliers on principal component plots) and 23 participants with missing phenotypic data leaving 435 placenta samples available for this analysis (see **Figure 1**).

#### Plasma

We prepared libraries using the Truseq Small RNA Sample Prep kit (Illumina, BC, Canada, catalog #RS-200-0012) following our standardized protocol detailed in (20). The quality of each library was evaluated using either the Agilent High Sensitivity DNA kit (Agilent, Mississauga, Ontario, Canada; catalog no 5067-4626) on the Agilent 2100 Bioanalyzer or the Kapa Illumina GA with Revised Primers-SYBR Fast Universal kit (Kapa Biosystems; library concentration) and the LabChip GX instrument (PerkinElmer, catalog no CLS760672; library length and absence of primer dimers). The libraries sent for sequencing were selected based on availability of samples and offspring longitudinal data (see **Figure 1**). Libraries (n=456) were sequenced at McGill University and Génome Québec Innovation Centre (Montréal, Canada) either on a HiSeq 2500 or HiSeq 4000 platform at 50 cycles, with 7 cycles indexing read. Twelve samples were sequenced twice on both platforms (Pearson’s correlation coefficient between platform ≥0.94). We removed the 12 duplicated samples, 7 outlier samples with low read counts (<500 000), and 15 participants with missing phenotypic data, leaving 422 plasma samples available for this analysis (see **Figure 1**).

### Statistical analyses

We visually inspected histograms of each continuous variable to assess distribution. We transformed the Stumvoll first phase index (Box-Cox), and the Matsuda Index (log_2_) to approach a normal distribution.

First, we investigated the association between insulin secretion, estimated using the Stumvoll first-phase index, and GDM development. We performed logistic regression with GDM as the outcome and the z-score of the Box-Cox transformed Stumvoll first-phase estimate as the exposure. The regression model included maternal age at first trimester, first trimester BMI, and gravidity status as covariates.

To process miRNA expression data in both placenta and plasma samples, we applied the extracellular RNA processing toolkit (exceRpt) pipeline (version 4.6) from Rozowsky et al. (22). Briefly, exceRpt uses FASTX-Toolkit and FastQC to remove the adapters and poor quality read (Phred score <20 for 80% or more of the read), applies STAR to align remaining high-quality sequences to the human genome (GRCh37) then performs miRNA quantification using miRbase (version 21) annotations. We removed miRNAs with low abundance by keeping only those with at least 6 read counts in a minimum of 20% of samples, leaving 674 miRNAs in plasma and 952 miRNAs in placenta for analyses. We normalized the retained miRNAs using the edgeR R package (23) by computing counts per million reads (CPM).

For the placenta miRNA data analyses, we adjusted models for Matsuda Index (log_2_), gravidity status, maternal age, newborn sex and gestational age at delivery as well as 5 surrogate variables (SVs) to account for cell type composition and technical bias including batch effects. We used the EstDimRMT function from isva (24) on the residual matrix - obtained by regressing the Stumvoll first phase estimate and adjustment variables on the miRNA counts - to determine the optimal number of SVs to estimate, which was found to be 21 based on the variance structure of our dataset. Subsequently, we applied the SmartSVA (25) R package to compute the 21 SVs, of which we retained only the first 5 for downstream analyses, as the remaining SVs captured biological variance. In the plasma miRNA analyses, we adjusted models for Matsuda Index (log_2_), gravidity status, maternal age and gestational age at first trimester (i.e., at the time of plasma collection for miRNA quantification), batch, and sequencing run. We also conducted sensitivity analyses to additionally include delivery mode as covariate. For both placenta and plasma analysis, we applied the voom (26) transformation to the CPM expression and then fit a linear model for each miRNA using Limma (27) identify differential levels of miRNAs with Stumvoll first phase index as a continuous independent variable; we reported miRNAs with differential levels in relation to the Stumvoll with *P*-values <0.05.

To further assess the robustness of these findings in the placenta and plasma datasets, we performed a bootstrap resampling analysis (28). In the placenta, considering the stability of the SVs, which capture batch effects and cell type composition, as well as the substantial computational burden associated with recomputing SVs in each bootstrap sample, we retained the 5 SVs estimated in the main analysis rather than recalculating them for each resampled dataset. For each miRNA, we reported the frequency of significance (*P*-value < 0.05) across bootstrap re-samples, as well as the 95% confidence interval (CI) around the normalized read count and beta values, corresponding to the values of the 2.5th and 97.5th percentiles of the distribution of the corresponding estimates across 1,000 bootstrap re-samples of the dataset. We did not correct for multiple testing but instead focused on insulin secretion-associated miRNAs identified in both plasma and placenta analyses and on direction of associations. Statistical analyses were conducted in R (version 4.0.3).

### miRNA-mRNA interactions

For miRNA associated with Stumvoll index in both the placenta and first trimester plasma analyses (same direction of association), we assessed miRNA-mRNA interactions using the DIANA database TarBase-v9.0 as it considers those validated experimentally (29). We applied the following filter: experimental throughput set to low, and experimental type including both direct and indirect methods.

### Data and resource availability

The datasets analyzed during the current study are available on dbGAP, (https://www.ncbi.nlm.nih.gov/projects/gap/cgi-bin/study.cgi?study_id=phs003151.v1.p1) and on GEO, (https://www.ncbi.nlm.nih.gov/geo/query/acc.cgi). No applicable resources were generated or analyzed during the current study.

## Results

### Participants’ characteristics

The characteristics of the 435 participants included in the placenta sample analyses are described in **Table 1**. Overall, women had a median [interquartile range (IQR)] age of 28.5 [25.4 - 31.5] years, and BMI of 23.8 [21.4 – 28.0] kg/m^2^ in the first trimester of pregnancy (gestational age: 9.3 [8.1 - 11.6] weeks); 159 (36.5%) were primigravid. In the second trimester of pregnancy (gestational age: 26.3 [25.9 - 27.3] weeks), women had a Stumvoll first phase index of 1112.9 [905.4 - 1284.5] and Matsuda index of 6.8 [4.7 – 9.4]; 36 (8.3%) met diagnosis criteria for GDM. The gestational age at delivery was 39.6 [38.7 - 40.3] weeks) and 205 (47.1%) of newborns were female. The characteristics of the 422 participants included in the analysis of miRNAs from plasma samples were very similar and are described in **Supplementary Table 1**.

**Table 1:** Characteristics of the 435 Gen3G participants with decidual/placental miRNA measurements.

Characteristic	Median [IQR] or n (%)
First trimester of pregnancy	
Age (years)	28.5 [25.4-31.5]
BMI (kg/m^2^)	23.8 [21.4-28.0]
Primigravid	159 (36.6)
Gestational age (weeks)	9.3 [8.1-11.6]
Second trimester of pregnancy	
Gestational age (week)	26.3 [25.9-27.3]
Diagnosis of GDM*	36 (8.3)
Stumvoll first phase index	1112.9 [905.4-1284.5]
Matsuda Index	6.8 [4.7-9.4]
Delivery	
Cesarian section	73 (16.8)
Gestational age (weeks)	39.6 [38.6-40.3]
Child’s sex (female)	205 (47.1)

*By the International Association of the Diabetes in Pregnancy Study Groups criteria. BMI, body mass index; GDM, gestational diabetes mellitus.

### Insulin secretion estimated by the Stumvoll index measured at ~26 weeks of gestation is associated with GDM status

Using logistic regression and controlling for maternal first-trimester age, first-trimester BMI, and gravidity, we found that higher insulin secretion, estimated using the z-score of the Box-Cox transformed Stumvoll first-phase index, measured at ~26 weeks’ gestation, was significantly associated with a lower odds of GDM at the same assessment (odds ratio [OR] = 0.56 per SD increase of Stumvoll, 95% confidence interval [CI]: 0.39-0.80, p-value=0.001).

### Decidual/placental and first trimester plasma miRNAs associated with insulin secretion estimated by the Stumvoll index measured at ~26 weeks of gestation

We detected a total of 952 miRNAs in placental samples and 674 miRNAs in first trimester plasma samples. We identified 30 decidual/placental and 93 first trimester plasma miRNAs nominally associated (*P*<0.05; no correction for multiple testing) with Stumvoll first phase index measured at ~26 weeks of gestation. Of these, 13 decidual/placental and 44 plasma miRNAs were positively associated with the Stumvoll index. **Supplementary Tables 2, 3** provide the full list of these miRNAs with their mean normalized read counts, percentages of placenta and plasma samples in which they were detected, beta, nominal *P*-values, and summary metrics from the bootstrap analysis. Briefly, the beta values, representing log2-fold changes in miRNA levels per unit increase in the Box-Cox transformed Stumvoll index, ranged from –9.34 to 7.65 in placental samples and from –14.0 to 14.9 in first trimester plasma samples. On average, the 30 decidual/placental miRNAs and 93 first trimester plasma miRNAs, remained significant in 95% and 92% of bootstrap iterations, respectively. Two of these miRNAs (hsa-miR-199a-3p|hsa-miR-199b-3p and hsa-miR-3150b-3 miRNAs) were nominally associated (*P*<0.05 and same direction of association) with the Stumvoll first phase index in both placenta and first trimester plasma samples (see **Table 2**). Specifically, lower insulin secretion was associated with lower levels of has-miR-199a-3p|has-miR-199b-3p (b=1.47 [0.11, 2.84] in placenta and b= 4.22 [0.93, 7.52] in plasma), and with higher levels of has-miR-3150b-3p (b= -6.97 [-13.85, -0.09] in placenta and b= -9.19 [-17.42, -0.96] in plasma). In our sensitivity analyses including delivery mode as a covariate, results remained essentially the same for hsa-miR-199a-3p|hsa-miR-199b-3p and hsa-miR-3150b-3 miRNAs.

**Table 2:** Common miRNAs associated with Stumvoll first phase index (*P*<0.05) in both placenta and first trimester plasma samples.

miRNA	Decidual/placental miRNA				Plasma miRNA			
	Beta (95% CI)	*P*-values	Mean normalized read count (95%IC) (log_2_CPM)	Detection rate (%)*	Beta (95% CI)	*P*-values	Mean normalized read count (95%CI) (log_2_CPM)	Detection rate (%)*
hsa-miR-199a-3p| hsa-miR-199b-3p	1.47 (0.10 to 2.69)	0.03	13.85 (13.80 to 13.89)	100.0	4.22 (0.70 to 7.67)	0.01	11.21 (11.11 to 11.31)	97.0
hsa-miR-3150b-3p	-6.97 (-14.39 to 0.40)	0.05	-2.54 (-2.66 to -2.43)	90.8	-9.19 (-18.39 to -0.60)	0.03	-0.76 (-0.87 to -0.64)	80.2

*Percentage of samples in which the listed miRNA was detected. Beta represents changes in log2 miRNA levels per unit increase in Box-Cox transformed Stumvoll estimate value.

### miRNA-mRNA interactions for miRNAs associated with the Stumvoll index in both the placenta and first trimester plasma analyses

**Table 3** present target genes of miRNAs associated with the Stumvoll index in both the placenta and first trimester plasma analyses. We identified miRNA–mRNA interactions only for hsa-miR-199a-3p, which targets the following genes: *DNAJA4, FLT1, TAB3, TGFB1*, and *ZHX1.*

**Table 3:** Experimentally validated microRNA–target gene interactions and associated experimental details.

miRNA	Gene^a^	Tissue	Cell line	Cell type	Experimental method	Experimental type	Micro tscore^b^
hsa-miR-199a-3p	*DNAJA4*	Skin	MEWO	NA	Luciferase Reporter Assay	Direct	0.57
	*FLT1*	Lung	A549	Epithelial cells			1
	*TAB3*	Liver	HCC Cell Line	NA			0.8
	*TGFB1*	Lung	HFL1	Fibroblasts	qPCR	Indirect	0.7
	*ZHX1*	Gastric	SGC7901	NA	Luciferase Reporter Assay	Direct	-1
hsa-miR-199b-3p	NA	NA	NA	NA	NA	NA	NA
hsa-miR-3150b-3	NA	NA	NA	NA	NA	NA	NA

a Experimentally validated interactions with miRNAs as retrieved from TarBase v9.0.

b Represents a confidence score reflecting the strength or reliability of each miRNA–gene interaction, with higher values indicating stronger support.

## Discussion

In this large prospective cohort, we used genome-wide miRNA sequencing to identify decidual/placental miARNs associated with insulin secretion during pregnancy. Our study identified 30 decidual/placental miRNAs suggestively associated (nominal *P*-value) with insulin secretion as estimated by the Stumvoll first phase index from 75g-OGTT performed at ~26 weeks of gestation. Two of these identified miRNAs (hsa-miR-199a-3p|hsa-miR-199b-3p, hsa-miR-3150b-3p) were detected in first trimester plasma samples and their circulating levels were associated with insulin secretion in later pregnancy. Although none of the associations would remain statistically significant after correction for multiple testing (e.g. FDR), we prioritized miRNAs that exhibited consistent directional associations across both placental and plasma tissues. As placental miRNAs are produced in trophoblast cells, and can be exported to maternal circulation (30), this consistency may reinforce their physiological relevance. Supporting this, a previous study reported that the ten most abundant miRNAs in mid-gestation placental tissue were also highly abundant in matched maternal plasma samples collected at the same time point (31).

Higher insulin secretion in pregnancy was associated with greater levels of hsa-miR-199a-3p|hsa-miR-199b-3p, in both placenta and plasma. These results are consistent with a previous study conducted by our group, which reported that hsa-miR-199a-3|hsa-miR-199b-3p were detected at lower levels in first-trimester plasma samples from women who later developed GDM, compared to those who did not (32). However, these miRNAs did not appear as GDM predictors in that previous study which may be because the analyses were conducted on the general GDM definition without any subtyping based on the underlying physiological processes (insulin resistance or deficiency). Given that our samples were collected from the maternal side of the placenta, it could be hypothesis that the release of hsa-miR-199a-3p|hsa-miR-199b-3p from decidual cells into the maternal circulation enhance insulin secretion during pregnancy and might protect against development of GDM. Further analyses should be conducted to investigate whether these miRNAs would be good predictors of GDM development in subtypes affected by insulin deficiency.

Hsa-miR-199a-3p|hsa-miR-199b-3p are expressed in multiple human tissues, including the pancreas (33). They are encoded in two distinct genes (MIR199a on chromosome 1 and MIR199b on chromosome 9, respectively), although their mature miRNAs are identical, allowing them to target the same mRNAs and exert similar post-transcriptional regulatory functions. Nevertheless, their distinct genomic location may have differential transcriptional regulation. To date, research has primarily focused on the biological functions of hsa-miR-199a-3p, particularly in the context of cancer (34) and only a few studies related to glycemic/insulin regulation. In the field of diabetes, Zhang et al. reported that hsa-miR-199a-3p was the most downregulated miRNA in peripheral blood from patients with diabetic nephropathy, and that its blood levels were negatively correlated with proteinuria in these patients (35). Using human proximal tubular kidney HK-2 cells, the authors demonstrated that hsa-miR-199a-3p inhibited apoptosis and the inflammatory response by targeting the IKKβ/NF-κB pathway *in vitro* (35). Another functional study in HK-2 kidney cells examining the role of hsa-miR-199a-3p in diabetic nephropathy showed that hsa-miR-199a-3p protects these cells from diabetic-induced injury by upregulating E-cadherin expression through the repression of KDM6A, a histone lysine demethylase (36). Interestingly, cadherins have been shown to directly influence the ability of pancreatic beta cells to secrete insulin in response to glucose by facilitating the formation of adhesion junctions (37). Moreover, hsa-miR-199a-3p has been shown to activate the Notch signaling pathway by inducing the overexpression of *Notch1, Jagged1, DII-1*, and *Hes1* genes in cultured cardiospheres (38). The Notch signaling pathway is expressed in the adult human pancreas, where it regulates beta cell proliferation and maturation (39). Inhibition of Notch1 has also been shown to reduce insulin secretion and beta cell mass in isolated pancreatic beta cells (40). Given these findings, the effects of hsa-miR-199a-3p on the regulation of E-cadherin and the Notch signaling pathway should be further explored in pancreatic beta cells and to determine whether these mechanisms are relevant to insulin secretion.

Higher levels of hsa-miR-3150b-3p (in both placenta and plasma) were associated with lower insulin secretion during pregnancy. To our knowledge, no study has yet investigated the role and actions of hsa-miR-3150b-3p in the context of pregnancy or diabetes. However, bioinformatic analyses suggest that hsa-miR-3150b-3p interacts with phospholipase A2 (41). Interestingly, inhibition of phospholipase A2 reduces glucose-induced insulin secretion in isolated human islets (42). Therefore, the downregulation of phospholipase A2 by hsa-miR-3150b-3p may represent a mechanism contributing to the association with reduced insulin secretion observed in our study, but this hypothesis needs to be tested in functional experiments.

### Strengths and limitations

The strengths of this study include a large sample size and the use of agnostic transcriptome-wide small RNA sequencing in two tissues from the same participant to identify novel insulin secretion-associated miRNAs. This study also has some limitations. First, we focused our attention on consistency of association (direction and nominal *P*<0.05) in miRNA datasets from two different tissues; however, we did not adjust for multiple hypothesis testing, with remaining possibility of false positives. We also lacked external validation in a different cohort. Thus, our findings should be interpreted with caution, replicated in other cohorts and be viewed as hypothesis-generating to be tested in future functional studies. In addition, our study is observational, so we cannot infer causality of the observed associations. Finally, our sample included only participants of European descent, which precludes the extrapolation of our results to other ancestries.

## Conclusion

In conclusion, we reported that lower insulin secretion in late second trimester of pregnancy was nominally associated with lower hsa-miR-199a-3p|hsa-miR-199b-3p and with higher hsa-miR-3150b-3p in both first trimester plasma and placenta at delivery. Our previous findings reported lower levels of hsa-mir-199a-3p|hsa-mir-199b-3p in the first-trimester plasma of pregnant women who later developed GDM, highlighting their potential protective role. The effects of hsa-mir-199a-3p on E-cadherin and the Notch signaling pathway should be further explored in pancreatic beta cells to determine whether these mechanisms are relevant to insulin secretion during pregnancy. These novel miRNAs may complement existing metabolic or genetic risk models to improve early prediction of GDM risk or be targeted to augment insulin secretion in GDM with insulin deficiency.

## Data Availability

The datasets presented in this study can be found in online repositories. The names of the repository/repositories and accession number(s) can be found in the article/**Supplementary Material**.
