# Supplementary material for: Decidual/placental and first trimester plasma levels of hsa-miR-199a-3p|hsa-miR-199b-3p and hsa-miR-3150b-3p are associated with insulin secretion in pregnancy
Source: Front Endocrinol (Lausanne). 2025 Aug 19;16:1622500. doi: 10.3389/fendo.2025.1622500 (PMC12401689; doi:10.3389/fendo.2025.1622500)
Supplement: Supplementary file 1 [file DataSheet1.docx]

**Supplement**

**Supplementary Table 1.** Characteristics of the 422 Gen3G participants with first trimester plasma miRNA measurements.

|  | Median [IQR] or n (%) |
| --- | --- |
| First trimester of pregnancy | |
| Age (years) | 28.9 [26.0 - 31.8] |
| BMI (kg/m^2^) | 24.2 [21.6 - 28.8] |
| Primigravid | 156 (37.0) |
| Gestational age (weeks) | 9.3 [8.1 - 11.6] |
| Second trimester of pregnancy | |
| Gestational age (week) | 26.3 [25.9 - 27.1] |
| Diagnosis of GDM* | 56 (13.3) |
| Stumvoll first phase index | 1118.3 [914.5 - 1298.5] |
| Matsuda Index | 6.27 [4.41 - 9.11] |
| Delivery | |
| Cesarian section | 74 (17.5) |
| Gestational age (weeks) | 39.6 [38.6 - 40.3] |
| Child’s sex (female) | 210 (49.8) |

Notes: *By the International Association of the Diabetes in Pregnancy Study Groups criteria. Abbreviations: BMI, body mass index; GDM, gestational diabetes mellitus.

**Supplementary Table 2.** Full list of decidual/placental miRNAs associated (nominal *P*-value) with insulin secretion (Stumvoll first phase estimate) during pregnancy.

| miRNAs | Normalized read count (95% CI) (log_2_CPM) | Detection rate^a^ | Beta (95% CI) | *P*-value | Bootstrap sign. freq. (p<0.05)^b^ |
| --- | --- | --- | --- | --- | --- |
| hsa-miR-624-3p | -0.36 (-0.42 to -0.30) | 100 | -4.40 (-7.33 to -0.93) | 6.67E-03 | 97 |
| hsa-miR-34a-3p | 2.71 (2.66 to 2.75) | 100 | -3.31 (-5.43 to -1.13) | 8.57E-03 | 98 |
| hsa-miR-196b-5p | 7.65 (7.60 to 7.70) | 100 | 2.38 (0.44 to 4.29) | 9.19E-03 | 96 |
| hsa-miR-152-3p | 9.31 (9.26 to 9.36) | 100 | 2.03 (0.57 to 3.45) | 0.01 | 98 |
| hsa-miR-24-2-5p | 1.88 (1. 84 to 1.92) | 100 | -2.41 (-4.46 to -0.64) | 0.01 | 97 |
| hsa-miR-3136-5p | -0.03 (-0.077 to 0.016) | 100 | -3.43 (-6.01 to -1.12) | 0.01 | 98 |
| hsa-miR-3160-3p | -1.95 (-2.05 to -1.87) | 97.7 | -6.53 (-10.98 to -1.86) | 0.01 | 98 |
| hsa-miR-548e-3p | 1.22 (1.17 to 1.26) | 100 | 3.17 (0.70 to 5.73) | 0.01 | 97 |
| hsa-miR-6726-3p | -1.36 (-1.46 to -1.27) | 98.4 | -6.47 (-10.72 to -1.83) | 0.01 | 98 |
| hsa-miR-190a-3p | -2.55 (-2.67 to -2.44) | 89.9 | 7.57 (2.23 to 13.55) | 0.02 | 98 |
| hsa-miR-190a-5p | 6.83 (6.78 to 6.88) | 100 | 2.76 (0.47 to 4.96) | 0.02 | 94 |
| hsa-miR-380-3p | 0.43 (0.36 to 0.49) | 100 | 3.11 (0.80 to 5.60) | 0.02 | 97 |
| hsa-miR-6501-3p | -1.38 (-1.47 to -1.28) | 98.2 | -5.92 (-9.91 to -1.75) | 0.02 | 98 |
| hsa-miR-6780b-3p | -0.58 (-0.64 to -0.51) | 100 | -4.43 (-7.74 to -0.91) | 0.02 | 96 |
| hsa-miR-135a-5p | 4.40 (4.25 to 4.53) | 100 | -9.34 (-17.52 to -1.30) | 0.03 | 94 |
| hsa-miR-199a-3p\|hsa-miR-199b-3p | 13.85 (13.80 to 13.89) | 100 | 1.47 (0.10 to 2.69) | 0.03 | 93 |
| hsa-miR-200c-5p | 5.02 (5.00 to 5.05) | 100 | -1.46 (-2.82 to -0.31) | 0.03 | 95 |
| hsa-miR-214-5p | 3.94 (3.89 to 4.00) | 100 | 1.93 (0.29 to 3.56) | 0.03 | 95 |
| hsa-miR-6513-3p | 2.38 (2.36 to 2.40) | 100 | -1.49 (-2.88 to -0.24) | 0.03 | 94 |
| hsa-miR-652-5p | 0.9 (0.86 to 0.95) | 100 | 3.07 (0.68 to 5.35) | 0.03 | 97 |
| hsa-miR-1283 | 12.14 (12.09 to 12.18) | 100 | -2.70 (-5.34 to -0.63) | 0.04 | 97 |
| hsa-miR-3139 | -1.11 (-1.18 to -1.04) | 99.8 | -4.13 (-8.3 to -0.34) | 0.04 | 93 |
| hsa-miR-6827-3p | -1.79 (-1.88 to -1.70) | 98.2 | -5.20 (-9.72 to -0.11) | 0.04 | 92 |
| hsa-miR-873-5p | 1.45 (1.38 to 1.52) | 100 | 4.10 (0.95 to 7.09) | 0.04 | 95 |
| hsa-miR-191-3p | 2.81 (2.77 to 2.84) | 100 | -1.88 (-3.80 to -0.16) | 0.05 | 93 |
| hsa-miR-191-5p | 12.76 (12.74 to 12.78) | 100 | -1.24 (-2.49 to -0.14) | 0.05 | 93 |
| hsa-miR-3150b-3p | -2.54 (-2.66 to -2.43) | 90.8 | -6.97 (-14.39 to 0.40) | 0.05 | 88 |
| hsa-miR-4719 | 0.63 (0.52 to 0.73) | 99.1 | 6.71 (-0.05 to 12.94) | 0.05 | 91 |
| hsa-miR-5187-5p | -0.36 (-0.41 to -0.31) | 100 | 3.16 (0.18 to 6.17) | 0.05 | 92 |
| hsa-miR-642a-5p | -1.02 (-1.17 to -0.86) | 97.2 | 7.65 (-0.84 to 15.61) | 0.05 | 89 |

Note: ^a^Percentage of samples in which the listed miRNA was detected. ^b^Percentage of bootstrap resamples in which the listed miRNA reached statistical significance (*P*-value < 0.05). Beta represents changes in log_2_ miRNA levels per unit increase in box-cox transformed Stumvoll estimate value.

**Supplementary Table 3.** Full list of first trimester plasma miRNAs associated (nominal *P*-value) with insulin secretion (Stumvoll first phase estimate) during pregnancy.

| miRNAs | Normalized read count (95% CI) (log_2_CPM) | Detection rate^a^ | Beta (95% CI) | *P*-value | Bootstrap sign. freq. (p<0.05)^b^ |
| --- | --- | --- | --- | --- | --- |
| hsa-miR-152-5p | 0.98 (0.86 to 1.12) | 94 | 11.05 (3.97 to 18.00) | 4.49E-04 | 99 |
| hsa-miR-150-3p | 3.20 (3.12 to 3.28) | 97 | -11.59 (-18.00 to -4.83) | 5.33E-04 | 100 |
| hsa-miR-3920 | -0.90 (-1.06 to -0.76) | 79.1 | 13.84 (5.29 to 21.79) | 5.43E-04 | 99 |
| hsa-miR-2355-3p | 1.13 (1.01 to 1.25) | 95.4 | 9.82 (3.03 to 16.42) | 7.21E-04 | 99 |
| hsa-miR-659-5p | 1.43 (1.35 to 1.51) | 96.1 | -7.98 (-12.46 to -3.78) | 8.75E-04 | 100 |
| hsa-miR-4433b-3p | 2.29 (2.12 to 2.45) | 97 | 14.89 (5.83 to 23.88) | 9.75E-04 | 100 |
| hsa-miR-33a-5p | 4.01 (3.90 to 4.12) | 97 | 6.20 (2.34 to 10.07) | 1.95E-03 | 99 |
| hsa-miR-7706 | 4.79 (4.75 to 4.83) | 97 | -6.40 (-11.46 to -1.53) | 2.83E-03 | 98 |
| hsa-miR-491-5p | -0.66 (-0.83 to -0.51) | 82.1 | 12.81 (3.54 to 21.83) | 3.62E-03 | 98 |
| hsa-miR-151a-5p\|hsa-miR-151b | 10.71 (10.62 to 10.80) | 97 | 4.39 (0.90 to 7.68) | 3.77E-03 | 97 |
| hsa-miR-577 | 1.44 (1.31 to 1.57) | 94.9 | 8.10 (2.96 to 13.31) | 4.08E-03 | 99 |
| hsa-miR-6868-3p | -0.56 (-0.72 to -0.42) | 78.2 | -14.04 (-24.07 to -3.59) | 4.26E-03 | 98 |
| hsa-miR-378a-3p | 9.84 (9.79 to 9.88) | 97 | -4.33 (-7.78 to -1.02) | 4.54E-03 | 97 |
| hsa-miR-199a-5p | 7.22 (7.11 to 7.32) | 97 | 5.18 (1.55 to 8.61) | 4.85E-03 | 98 |
| hsa-miR-1255a | 2.32 (2.17 to 2.47) | 94.7 | 12.84 (2.87 to 22.18) | 5.52E-03 | 97 |
| hsa-miR-501-3p | 2.86 (2.81 to 2.92) | 97 | -5.52 (-9.12 to -1.91) | 5.53E-03 | 99 |
| hsa-miR-26b-3p | 6.28 (6.20 to 6.37) | 97 | 3.69 (0.85 to 6.45) | 5.81E-03 | 97 |
| hsa-miR-1306-3p | 0.88 (0.77 to 0.98) | 95.9 | 7.84 (1.13 to 14.70) | 5.88E-03 | 96 |
| hsa-miR-628-5p | 3.52 (3.42 to 3.62) | 97 | 5.20 (1.37 to 9.06) | 6.70E-03 | 98 |
| hsa-miR-145-5p | 4.03 (3.91 to 4.14) | 97 | 6.82 (2.04 to 11.73) | 7.02E-03 | 98 |
| hsa-miR-423-5p | 13.97 (13.93 to 14.01) | 97 | -4.44 (-7.94 to -0.83) | 7.29E-03 | 96 |
| hsa-miR-579-5p | -0.65 (-0.77 to -0.52) | 82.8 | 11.02 (2.29 to 19.05) | 8.00E-03 | 97 |
| hsa-miR-486-5p | 18.47 (18.40 to 18.52) | 97 | -8.93 (-15.66 to -1.88) | 8.29E-03 | 97 |
| hsa-miR-28-5p | 7.94 (7.85 to 8.03) | 97 | 3.62 (0.61 to 6.54) | 8.79E-03 | 95 |
| hsa-miR-126-5p | 13.85 (13.76 to 13.93) | 97 | 3.45 (0.31 to 6.31) | 0.01 | 95 |
| hsa-miR-199a-3p\|hsa-miR-199b-3p | 11.21 (11.11 to 11.31) | 97 | 4.22 (0.70 to 7.67) | 0.01 | 96 |
| hsa-miR-19b-3p | 9.77 (9.72 to 9.81) | 97 | -4.10 (-8.10 to -0.39) | 0.01 | 94 |
| hsa-miR-25-3p | 13.21 (13.17 to 13.25) | 97 | -6.58 (-11.63 to -1.02) | 0.01 | 95 |
| hsa-miR-3127-5p | 1.37 (1.30 to 1.45) | 97 | -6.19 (-11.08 to -0.84) | 0.01 | 95 |
| hsa-miR-378c | 0.53 (0.43 to 0.63) | 94.5 | -8.33 (-15.37 to -1.23) | 0.01 | 95 |
| hsa-miR-378e | -0.49 (-0.59 to -0.40) | 90.1 | -8.01 (-14.74 to -2.18) | 0.01 | 98 |
| hsa-miR-6889-3p | -0.96 (-1.08 to -0.83) | 78.2 | 10.43 (2.02 to 19.63) | 0.01 | 96 |
| hsa-miR-101-3p | 12.64 (12.59 to 12.69) | 97 | -4.62 (-9.44 to -0.27) | 0.02 | 93 |
| hsa-miR-1185-5p | -0.03 (-0.20 to 0.14) | 83.9 | 11.43 (0.36 to 22.69) | 0.02 | 93 |
| hsa-miR-126-3p | 10.84 (10.76 to 10.92) | 97 | 2.47 (0.08 to 4.72) | 0.02 | 91 |
| hsa-miR-130b-3p | 8.59 (8.55 to 8.63) | 97 | -2.86 (-5.32 to -0.41) | 0.02 | 95 |
| hsa-miR-133a-3p | 4.41 (4.26 to 4.55) | 96.8 | 8.29 (2.11 to 14.38) | 0.02 | 96 |
| hsa-miR-151a-5p | 5.16 (5.05 to 5.26) | 97 | 4.89 (0.52 to 8.85) | 0.02 | 94 |
| hsa-miR-181b-3p | 0.89 (0.77 to 1.01) | 94.3 | -7.34 (-13.51 to -1.55) | 0.02 | 96 |
| hsa-miR-181c-5p | 8.55 (8.46 to 8.64) | 97 | 3.10 (0.66 to 5.48) | 0.02 | 96 |
| hsa-miR-181d-5p | 6.75 (6.66 to 6.84) | 97 | 3.77 (0.43 to 7.00) | 0.02 | 94 |
| hsa-miR-25-5p | 3.41 (3.36 to 3.47) | 97 | -5.31 (-9.75 to -0.84) | 0.02 | 95 |
| hsa-miR-301b-3p | 5.47 (5.38 to 5.56) | 97 | 3.47 (0.59 to 6.22) | 0.02 | 95 |
| hsa-miR-3615 | 8.41 (8.37 to 8.45) | 97 | -3.74 (-7.20 to -0.36) | 0.02 | 94 |
| hsa-miR-362-3p | 0.91 (0.80 to 1.01) | 93.3 | -9.06 (-16.78 to -1.22) | 0.02 | 94 |
| hsa-miR-424-3p | 5.60 (5.54 to 5.66) | 97 | -6.72 (-12.72 to -0.55) | 0.02 | 94 |
| hsa-miR-4732-5p | 3.90 (3.81 to 4.00) | 97 | -10.17 (-18.24 to -1.45) | 0.02 | 95 |
| hsa-miR-6837-3p | 2.70 (2.60 to 2.80) | 97 | 5.39 (0.66 to 10.24) | 0.02 | 93 |
| hsa-miR-107 | 12.27 (12.21 to 12.32) | 97 | -6.47 (-12.29 to -0.11) | 0.03 | 91 |
| hsa-miR-10b-5p | 12.20 (12.13 to 12.27) | 97 | -6.32 (-12.31 to -0.37) | 0.03 | 93 |
| hsa-miR-1306-5p | 2.00 (1.91 to 2.09) | 97 | -5.31 (-9.94 to -0.88) | 0.03 | 95 |
| hsa-miR-142-3p | 8.63 (8.52 to 8.75) | 97 | 4.01 (0.60 to 7.67) | 0.03 | 94 |
| hsa-miR-182-5p | 11.79 (11.73 to 11.85) | 97 | -6.65 (-13.76 to 0.12) | 0.03 | 92 |
| hsa-miR-18a-3p | 4.92 (4.86 to 4.98) | 97 | -5.27 (-10.56 to 0.29) | 0.03 | 91 |
| hsa-miR-192-5p | 11.58 (11.53 to 11.64) | 97 | -5.82 (-11.66 to -0.40) | 0.03 | 93 |
| hsa-miR-3150b-3p | -0.76 (-0.87 to -0.64) | 80.2 | -9.19 (-18.39 to -0.60) | 0.03 | 93 |
| hsa-miR-320d | 2.77 (2.70 to 2.84) | 97 | -4.60 (-9.45 to 0.89) | 0.03 | 92 |
| hsa-miR-345-5p | 7.18 (7.11 to 7.23) | 97 | -3.07 (-6.30 to -0.13) | 0.03 | 90 |
| hsa-miR-4646-5p | 0.21 (0.10 to 0.32) | 92.2 | -7.28 (-13.95 to -0.74) | 0.03 | 94 |
| hsa-miR-495-3p | 1.46 (1.29 to 1.62) | 92.6 | 9.54 (0.77 to 18.37) | 0.03 | 95 |
| hsa-miR-532-5p | 8.46 (8.41 to 8.50) | 97 | -5.52 (-10.53 to -0.43) | 0.03 | 93 |
| hsa-miR-625-5p | 5.93 (5.81 to 6.06) | 97 | 5.92 (0.74 to 11.00) | 0.03 | 94 |
| hsa-miR-660-5p | 6.94 (6.88 to 6.99) | 97 | -5.36 (-10.54 to -0.27) | 0.03 | 93 |
| hsa-miR-98-5p | 10.74 (10.65 to 10.84) | 97 | 4.03 (-0.21 to 8.13) | 0.03 | 91 |
| hsa-miR-103a-3p | 13.29 (13.25 to 13.33) | 97 | -3.24 (-6.48 to 0.06) | 0.04 | 89 |
| hsa-miR-1301-3p | 3.61 (3.51 to 3.70) | 97 | 3.99 (0.07 to 8.52) | 0.04 | 90 |
| hsa-miR-15b-3p | 4.94 (4.86 to 5.00) | 97 | -5.73 (-11.44 to 0.42) | 0.04 | 90 |
| hsa-miR-199b-5p | 2.90 (2.81 to 2.99) | 97 | -3.44 (-6.73 to -0.30) | 0.04 | 92 |
| hsa-miR-200c-3p | 1.05 (0.92 to 1.17) | 95.9 | 6.27 (-0.01 to 12.63) | 0.04 | 90 |
| hsa-miR-2355-5p | 3.52 (3.41 to 3.61) | 97 | 4.09 (0.00 to 8.06) | 0.04 | 91 |
| hsa-miR-26a-5p | 14.89 (14.79 to 14.99) | 97 | 3.09 (-0.18 to 6.25) | 0.04 | 90 |
| hsa-miR-320a | 11.55 (11.48 to 11.61) | 97 | -2.72 (-6.06 to 0.81) | 0.04 | 89 |
| hsa-miR-331-3p | 4.69 (4.57 to 4.80) | 97 | 4.87 (0.16 to 10.08) | 0.04 | 92 |
| hsa-miR-376a-5p | 2.96 (2.78 to 3.13) | 95.2 | 8.55 (0.69 to 17.04) | 0.04 | 91 |
| hsa-miR-421 | 7.62 (7.57 to 7.66) | 97 | -1.95 (-4.42 to 0.22) | 0.04 | 89 |
| hsa-miR-485-3p | 4.67 (4.52 to 4.80) | 97 | 7.49 (0.18 to 15.10) | 0.04 | 90 |
| hsa-miR-574-5p | 3.66 (3.56 to 3.77) | 97 | 4.87 (0.28 to 9.76) | 0.04 | 93 |
| hsa-miR-584-5p | 11.01 (10.93 ro 11.09) | 97 | 3.30 (-0.26 to 6.53) | 0.04 | 91 |
| hsa-miR-641 | 2.40 (2.30 to 2.49) | 96.8 | 4.29 (-0.85 to 9.34) | 0.04 | 87 |
| hsa-miR-6511a-3p | 3.17 (3.10 to 3.25) | 97 | -5.30 (-10.34 to -0.45) | 0.04 | 93 |
| hsa-miR-760 | 2.43 (2.32 to 2.54) | 96.6 | 4.84 (-1.36 to 10.62) | 0.04 | 87 |
| hsa-miR-940 | -0.67 (-0.82 to -0.51) | 78.6 | 8.39 (0.32 to 16.50) | 0.04 | 91 |
| hsa-miR-942-5p | 5.06 (5.02 to 5.10) | 97 | -4.30 (-8.26 to -0.32) | 0.04 | 92 |
| hsa-miR-1185-1-3p | 2.82 (2.65 to 2.98) | 96.1 | 8.12 (0.55 to 16.22) | 0.05 | 92 |
| hsa-miR-143-5p | 0.32 (0.16 to 0.46) | 92.4 | 8.04 (-0.28 to 16.46) | 0.05 | 90 |
| hsa-miR-181a-5p | 14.12 (14.04 to 14.18) | 97 | -2.94 (-5.79 to -0.03) | 0.05 | 92 |
| hsa-miR-181c-3p | 6.63 (6.52 to 6.73) | 97 | 3.85 (0.16 to 7.76) | 0.05 | 89 |
| hsa-miR-2115-3p | -0.50 (-0.62 to -0.38) | 84.6 | -9.25 (-18.72 to 0.33) | 0.05 | 89 |
| hsa-miR-301a-3p | 8.85 (8.77 to 8.92) | 97 | 1.97 (-0.18 to 3.98) | 0.05 | 88 |
| hsa-miR-3065-3p | 1.89 (1.76 to 2.01) | 95.9 | 5.59 (-0,39 to 11.62) | 0.05 | 89 |
| hsa-miR-371b-5p | 0.58 (0.47 to 0.68) | 93.1 | -7.57 (-15.12 to 0.62) | 0.05 | 89 |
| hsa-miR-3939 | -0.68 (-0.81 to -0.56) | 82.8 | 7.49 (-1.96 to 17.29) | 0.05 | 85 |
| hsa-miR-92a-3p | 16.25 (16.22 to 16.28) | 97 | -2.84 (-5.92 to 0.12) | 0.05 | 91 |

Note: ^a^Percentage of samples in which the listed miRNA was detected. ^b^Percentage of bootstrap resamples in which the listed miRNA reached statistical significance (*P*-value < 0.05). Beta represents changes in log_2_ miRNA levels per unit increase in box-cox transformed Stumvoll estimate value.
